# Individual differences in visuo-spatial working memory capacity and prior knowledge during interrupted reading

**DOI:** 10.3389/fcogn.2024.1434642

**Published:** 2024-09-04

**Authors:** Francesca Zermiani, Prajit Dhar, Florian Strohm, Sibylle Baumbach, Andreas Bulling, Maria Wirzberger

**Affiliations:** ^1^University of Stuttgart, Institute of Educational Science, Department of Teaching and Learning with Intelligent Systems, Stuttgart, Germany; ^2^Department of Psychology, University of Potsdam, Potsdam, Germany; ^3^University of Stuttgart, Institute for Visualization and Interactive Systems, Department of Computer Science, Stuttgart, Germany; ^4^University of Stuttgart, Institute for Literary Studies, Department of English Literatures and Cultures, Stuttgart, Germany

**Keywords:** resumption, reading, interruption, visuo-spatial working memory, prior knowledge, individual differences

## Abstract

Interruptions are often pervasive and require attentional shifts from the primary task. Limited data are available on the factors influencing individuals' efficiency in resuming from interruptions during digital reading. The reported investigation—conducted using the InteRead dataset—examined whether individual differences in visuo-spatial working memory capacity (vsWMC) and prior knowledge could influence resumption lag times during interrupted reading. Participants' vsWMC capacity was assessed using the symmetry span (SSPAN) task, while a pre-test questionnaire targeted their background knowledge about the text. While reading an extract from a Sherlock Holmes story, they were interrupted six times and asked to answer an opinion question. Our analyses revealed that the interaction between vsWMC and prior knowledge significantly predicted the time needed to resume reading following an interruption. The results from our analyses are discussed in relation to theoretical frameworks of task resumption and current research in the field.

## 1 Introduction

Consider this scenario: you are reading an article on your computer for your research project. As you immerse yourself in the reading (primary task), you are notified that you have received an email about planning your upcoming business trip (secondary task). After carefully reading the instructions contained in the email, you need to refocus your attention on the article. Interruptions, acting as a pervasive source of distraction, frequently redirect our attention to secondary tasks, leading to negative consequences across different contexts. For instance, interruptions in health care can impair concentration levels and essential clinical information processing in physicians during daily attending rounds (Armendariz et al., 2021). Distractions have also often been linked to a decline in driving performance (Stutts et al., 2005). Moreover, frequent interruptions, such as those from social media and extensive messaging, can reduce overall productivity and negatively impact academic performance (Rosen et al., 2013).

Among educational contexts, reading can serve as both a source of entertainment and as a means of transmitting knowledge (Chevet et al., 2022c). As such it is a demanding task both to learn and teach (Wijekumar et al., 2012). The pervasive use of digital devices often results in interruptions during reading, diverting attention to alternative tasks and causing a shift from the ongoing activity to a new one (Chevet et al., 2022a). While numerous studies have analyzed the characteristics of interruptions and how disruptive they can be on performance (Trafton et al., 2005, 2003; Wirzberger and Russwinkel, 2015), there has been relatively less emphasis on specific individual differences influencing how we can efficiently recover from interruptions (Bai et al., 2014; Drews and Musters, 2015; Meys and Sanderson, 2013). Nevertheless, exploring the impact of such characteristics on interruption recovery during reading has the potential to deepen our understanding of the cognitive process of task resumption. Furthermore, it could fundamentally improve the design of adaptive learning systems and teaching practices for reading purposes.

Prior research on interruptions has mostly examined memory processes during resumption, with the Memory for Goals theory (Altmann and Trafton, 2002) being a prominent activation-based framework in this domain. Informed by the cognitive theory of adaptive control of thoughts-rational (ACT-R) (Anderson and Lebiere, 1998), this theory assumes that we allocate our attention and cognitive resources to the task with the most active goal representations in our memory. Altmann and Trafton (2002) attributed the disruption caused by an interruption to the time necessary to reactivate goal representations in our memory upon resuming the task. To measure resumption performance, Trafton et al. (2003) introduced the concept of resumption lag as the time required to re-focus on the primary task following an interruption. Prior research have identified predictors of resumption performance, particularly in procedural and emergency tasks (Bai et al., 2014; Drews and Musters, 2015; Foroughi et al., 2016b; Joslyn and Hunt, 1998; Seamster et al., 1993). Preliminary findings suggested a potential influence of individual differences in reading habits, comprehension and multitasking skills on resumption performance during reading (Altamura et al., 2022). Among others, Foroughi et al. (2016a) reported interruptions to have a detrimental effect on reading comprehension for subjects with lower WMC. Moreover, variations in WMC influenced the level of mind wandering and text comprehension during a reading task (Schurer and Schubert, 2019). Despite the acknowledged impact of such individual characteristics on interruption recovery, its extent remains unclear due to limited data (Bai et al., 2014; Drews and Musters, 2015; Meys and Sanderson, 2013), particularly for interrupted reading tasks.

Previous findings have highlighted the significance of spatial representations in task resumption, a perceptual process not covered by the Memory for Goals theory (Ratwani and Trafton, 2008; Cane et al., 2012). In visual search research, resuming an interrupted task was found to be faster than starting one, indicating that some spatial configurations of the search display was retained during interruptions (Lleras et al., 2005). In more complex tasks, findings showed inconsistencies, likely due to the variety of experimental tasks and tests and the exclusive use of reaction time as measurement of resumption lag. Higher mental rotation task scores were correlated with shorter resumption times in a video cassette recorder programming task interrupted by pursuit tracking, whereas paper folding task scores did not predict performance (Werner et al., 2011). Conversely, variations in spatial abilities measured by the Mental Rotations Test (Peters et al., 1995) did not impact resumption time or accuracy during arithmetic tasks interrupted by lexical decision tasks (Meys and Sanderson, 2013). However, prior work using eye-tracking data to explore resumption found that individuals can return accurately close to the last fixated area during a task requiring them to type odd numbers from a list, as evidenced by their fixation behavior (Ratwani and Trafton, 2008). Besides, interruptions involving mental rotation caused longer and less accurate resumption than arithmetic problems (Ratwani and Trafton, 2008).

During reading instead, previous research demonstrated that we rely to some extent on our limited memory for the location of words and information within the text (Inhoff and Weger, 2005; Rothkopf, 1971). In interrupted reading, shorter resumption lags were achieved by visually signaling the last word that was read before an interruption occurred (Cane et al., 2012). Specifically, readers spent significantly less time re-reading the pre-interruption text when the last word was highlighted, aligning with previous findings on the signaling effect in multimedia learning (Schneider et al., 2018). Jo et al. (2015) applied this support strategy to develop a gaze-based digital bookmarking tool, demonstrating significantly reduced resumption times in highlighted conditions compared to a non-highlighted one. Upon resumption, readers also frequently re-read portions of text they had already covered before the interruption, as indicated by their eye movements (Cane et al., 2012; Chevet et al., 2022a). Resumption during reading therefore involves not only reinstating previously read information in memory, but in particular locating the point of interruption in the text, hence drawing on spatial memory (Cane et al., 2012). However, while these studies pointed at an association between spatial memory and resumption time during reading (Cane et al., 2012), none of them employed psychometric measurements of spatial memory. Studies measuring spatial ability typically focused on skills like mental spatial manipulation and visualization, but remembering spatial configurations is likely more important for task resumption (Meys and Sanderson, 2013).

Representations from previous knowledge stored in long-term memory guide readers in the process of deriving meaning from texts (Kintsch and van Dijk, 1978; Van Dijk and Kintsch, 1983). As a result, prior knowledge functions as a good predictor of reading comprehension (Abdelaal and Sase, 2014). Readers with a greater prior knowledge about the text exhibited improved skills in filtering out irrelevant information, hence a better processing and understanding of the text (McCarthy et al., 2018; Schurer and Schubert, 2019).

Studies investigating prior knowledge in the context of interrupted reading remain limited. These studies defined prior knowledge simply as familiarity or non-familiarity with the content of the reading material, based on the participants' educational background, neglecting the multiple dimensions of prior knowledge in text comprehension (McCarthy and McNamara, 2021). Findings from McNamara and Kintsch (1996) showed that mid-sentence interruptions during unfamiliar and difficult paragraphs result in longer reading times per sentence, as opposed to familiar paragraphs. More recently, Chevet et al. (2022b) observed increased fixations per character when readers lack prior knowledge about an expository text, regardless of the presence of interruptions. Similarly, prior knowledge had no impact on readers' attention, measured as frequency of mind wandering episodes during reading of an expository hypertext (Schurer and Schubert, 2019).

Nevertheless, prior knowledge significantly enhances perceptual processing during learning, allowing for more efficient integration and retrieval of new information, which is critical for problem-solving and deeper comprehension (Chi et al., 1982; Schnotz, 2002). The link between prior knowledge and visuo-spatial processing has been indeed previously addressed in theoretical frameworks of multimedia learning. Readers construct multiple representations of a text (Schnotz, 2002). They form a surface representation with linguistic details. Moreover, they build a mental model—or situation model in narrative comprehension (Van Dijk and Kintsch, 1983)—that integrates text content with domain-specific background knowledge (Schnotz, 2002). Such models are therefore complex, multi-sensory representations that integrate information from visual perception, prior knowledge, and potentially other sensory inputs (Mayer, 1997; Schnotz, 2002). While previous research has found evidence of a relevant link between prior knowledge and visuo-spatial processing in text comprehension and learning (Schnotz, 2002; Taub and Azevedo, 2019), this relationship has yet to be explored in the context of interrupted reading.

The present study addresses the need for research on the association between spatial memory and resumption time during interrupted reading using a psychometric test of spatial memory. Additionally, this study tackles the limited data on the role of the multiple dimensions of prior knowledge during resumption in reading. It thus examines how variations in visuo-spatial working memory capacity (vsWMC) and prior knowledge influence resumption lags after interruptions, building upon the significance of these cognitive factors in learning and text comprehension. We specifically hypothesize that: (H1) individuals with higher vsWMC scores should exhibit lower resumption lags following an interruption during reading; (H2) individuals with higher scores in prior knowledge on the content of the text should exhibit lower resumption lags following an interruption during reading.

To test these hypotheses, we built on the existing InteRead dataset for interrupted reading (Zermiani et al., 2024), since it includes detailed annotations of resumption lag times, and comprehensive pre- and post-test measurements. In particular, pre-test measures provide scores for participants' vsWMC and prior knowledge regarding the reading material, consisting of a long fictional text. vsWMC was measured through a Symmetry Span (SSPAN) task, whereas prior knowledge was assessed using a customized questionnaire, targeting different dimensions such as the participants' familiarity with the specific genre and characteristics of the selected fiction (Zermiani et al., 2024). We thus analyzed the influence of the provided individual scores on the annotated resumption times. To determine if other reader-related factors affect resumption time, we also analyzed pre- and post-test measures known to influence reading behavior, such as reading habits—frequency and print vs. digital media (Altamura et al., 2022), reading comprehension (Chevet et al., 2022a), and perceived reading experience (Chevet et al., 2022c), which have been linked to attentional shifts and disruptions during reading. We present novel preliminary findings suggesting that the interaction of vsWMC and prior knowledge may be a stronger predictor of resumption performance during interrupted reading, compared to their effect as individual predictors.

## 2 Method

The InteRead dataset was designed for investigating the impact of interruptions on reading behavior (Zermiani et al., 2024). InteRead comprises eye-tracking data collected from 50 participants engaged in an interrupted reading task of an English fictional text. The interruptions were strategically introduced throughout the reading task to investigate how participants manage and resume their reading following such disruptions.

### 2.1 Dataset overview

A total of 50 adult participants (*M*_*age*_ = 27.78, *SD*_*age*_ = 5.65, 30 female) with no diagnosed attention or reading disorders and normal or corrected-to-normal eyesight were included in the in-person laboratory study underlying the chosen dataset. English proficiency was assessed to include only native speakers or those with C1 proficiency, demonstrated by an IELTS score of 6.5+ or an equivalent standard. Based on their reported educational background, the subjects can be broadly grouped into the following categories (with the number of subjects in parentheses): computer science (17), pedagogy (7), linguistics (6), architecture (5), English (3), mechanical engineering (3), psychology (2), music (2), bio-engineering (1), materials science (1), secondary education (1), and social science (1).

The data collection consisted of a pre-test phase, the interrupted reading task involving eye tracking, and a post-test phase. The pre-test questionnaire presented standard demographic questions, a prior knowledge questionnaire—designed to assess participants' background knowledge on Sherlock Holmes stories and their familiarity with the crime genre—as well as a SSPAN task. The prior knowledge questionnaire consisted of 12 items, with a reported overall scale reliability of α = 0.83 (Zermiani et al., 2024). The questionnaire targeted three dimensions: detailed knowledge about Sherlock Holmes stories, with multiple-choice questions (e.g., “Who is the author of Sherlock Holmes?” or “What is the name of Sherlock Holmes' landlady?”); target-domain knowledge about previous exposure to crime fiction and related media, with yes/no agreement statements [e.g., “I have recently read a detective/crime fiction story.” or “I have recently seen a film or a TV adaptation of Sherlock Holmes (for example, the BBC series Sherlock).”]; and general knowledge about participants' interest in the crime genre, with 6-point Likert scale agreement statements (e.g., “I usually recommend reading detective/crime fiction books.” or “When deciding for a new book to read, I often pick a detective/crime fiction story.”) (Zermiani et al., 2024). All items were summed up to achieve prior knowledge total scores. The SSPAN task (Kane et al., 2004; Shah et al., 1996) represents a vested approach to assess vsWMC. From the various possibilities for implementation, we reproduced Unsworth et al. (2009)'s version of the SSPAN, following the outlined paradigm design as closely as possible. In detail, as storage task, participants had to recall the sequence of colored squares in a 4 × 4 grid after each square was displayed for 650 ms. Additionally, a processing task required to judge the vertical symmetry of grids with black squares displaying different shapes. Participants completed three blocks of trials, with the number of colored squares to recall as well as symmetry judgments randomly varying from two to five per trial respectively (Unsworth et al., 2009). Following established procedures (Foster et al., 2015; Redick et al., 2012), the participants' partial recall scores and the symmetry accuracy were calculated from the SSPAN task (Zermiani et al., 2024). The partial recall score refers to the sum of the squares recalled in the correct position, regardless of whether the entire sequence of squares was recalled in the correct order. It ranges from 0 to 42. The symmetry accuracy corresponds to the proportion of correct symmetry judgments. Additionally, in the pre-test, participants answered two questions regarding their leisure reading frequency and their preferred reading medium (Zermiani et al., 2024).

For the interrupted reading task, participants were asked to read an excerpt (28 pages) from the Arthur Conan Doyle's Sherlock Holmes story “The Adventure of the Speckled Band.” Six fixed pages contained an interruption, consisting of a dialog box that hid the text and prompted participants to answer an opinion question within 60 seconds. The questions, unrelated to the story, focused on everyday situations and general debate topics and were selected from Pashler et al. (2013). Building on previous evidence related to the seductive detail effect (Harp and Mayer, 1998), which describes how extraneous but interesting information hampers attention and learning, the opinion questions aimed at ensuring an adequate scope of interrupting potential. The questions were delivered as text items, since seductive texts have been demonstrated to particularly capture participants' attention (Rey, 2012). Furthermore, to reduce the possibility for subvocal rehearsal of the page content, thereby engaging the phonological loop (Baddeley, 1992), the questions required participants to think deeply and provide written content. Such interactive interrupting task thus aimed to substantially occupy participants' cognitive resources and disrupt both verbal and visual processing, in line with Oulasvirta and Saariluoma (2006). Interruptions were triggered when subjects' eye movements hit a pre-established target word, following previous approaches (Cane et al., 2012; Jo et al., 2015). To measure task performance following the interruptions, Zermiani et al. (2024) obtained participants' resumption lags, defined as as the time interval between the end of an interruption to the first stable reading pattern in the pre-interruption text. Resumption lags were manually annotated from the gaze data by two human annotators using a visualization and annotation tool (Zermiani et al., 2024).

The post-test survey partially consisted of four multiple-choice reading comprehension questions, to ensure the participants had mindfully read the story, and six statements on their reading experience related to the experiment, rated on 6-point Likert scales. Such statements included four items from the story world absorption scale (Kuijpers et al., 2014) and an item for participants' level of annoyance toward the interruptions they encountered (Zermiani et al., 2024).

### 2.2 Scoring

While the above-described variables and their corresponding scores were already included in the dataset, we calculated additional scores based on the available data through supplementary procedures. Reading frequency in InteRead was assessed based on the selected option to the question “How often do you read for enjoyment? (0) Never, (1) 1–2 times a week, (2) 2–3 times a week, (3) 4–5 times a week, (4) Everyday” (Zermiani et al., 2024). After observing the data distribution for this question, we opted for categorizing participants into three frequency groups to obtain a well-balanced bins distribution: low-frequency readers—subjects who never read for enjoyment (*n* = 10); medium-frequency readers—subjects who read between one and three times per week for enjoyment (*n* = 29); high-frequency readers—subjects who read more than three times a week for enjoyment (*n* = 8). In addition, to obtain the score indicating the preferred reading medium, we categorized participants into print vs. digital reading medium, based on their selected answer to the question “What medium do you choose to read from?”: (0) Print book, (1) E-book, (2) Computer, (3) Smartphone, (4) Other (Zermiani et al., 2024).

For reading experience, we followed a standard approach, which considers the Likert scales as an approximately continuous variable (Johnson and Creech, 1983). To achieve that, we assigned scores to the extreme points of each Likert scale, thereby creating a continuous range of values. Consequently, participants' scores per Likert scale were transformed to values within this range. By combining all six Likert scales related to the questionnaire, we obtained an individual reading experience score for each participant on a continuous scale.

### 2.3 Statistical analyses

To investigate the proposed effects of vsWMC and prior knowledge on resumption times, we used a standard linear mixed-effects model building on the R *lme4* package^1^ (Bates et al., 2015) and the *lmerTest* package^2^ (Kuznetsova et al., 2017) to obtain *p*-values for fixed effects. Our model included resumption lags as criterion variable; and partial recall scores from the SSPAN task, prior knowledge scores, as well as their interaction as predictor variables. We included subject ID and page as random effects to account for multiple data points over different pages by the same participants, since each individual had, on average, six resumption lags, resulting in a total of 272 observations. All predictor variables were normalized.

In addition, we aimed to verify if other reader-related factors had influenced resumption time. We used a similar linear mixed-effects model for that purpose. In particular, the model had the resumption lags as criterion variable; the partial recall score, prior knowledge, their interaction, reading frequency, print vs. digital reading medium, reading comprehension and reading experience as predictor variables. The same random effects were included.

## 3 Results

To ensure that the processing component of the SSPAN task was adequately included, we opted for a data-driven approach and excluded participants who scored 2 SD points below the processing performance mean (Richmond et al., 2022). This resulted in the exclusion of three subjects, hence a total sample size of *n* = 47 (see Table 1 for more detailed descriptive statistics).

**Table 1:** Descriptive statistics for resumption lag, partial recall score, and prior knowledge.

	***M***	***SD***	***Min***	***Max***
Partial recall score (SSPAN)	27.57	7.60	5	41
Prior knowledge	11.21	6.65	0	24
Resumption lag (s)	2.81	1.54	0.95	8.29

Total sample size *n* = 47; *M*, mean; *SD*, standard deviation; *Min*, minimum; *Max*, maximum; s, seconds; *partial recall score* as measurement of vsWMC follows (Redick et al., 2012).

**Figure 1:**
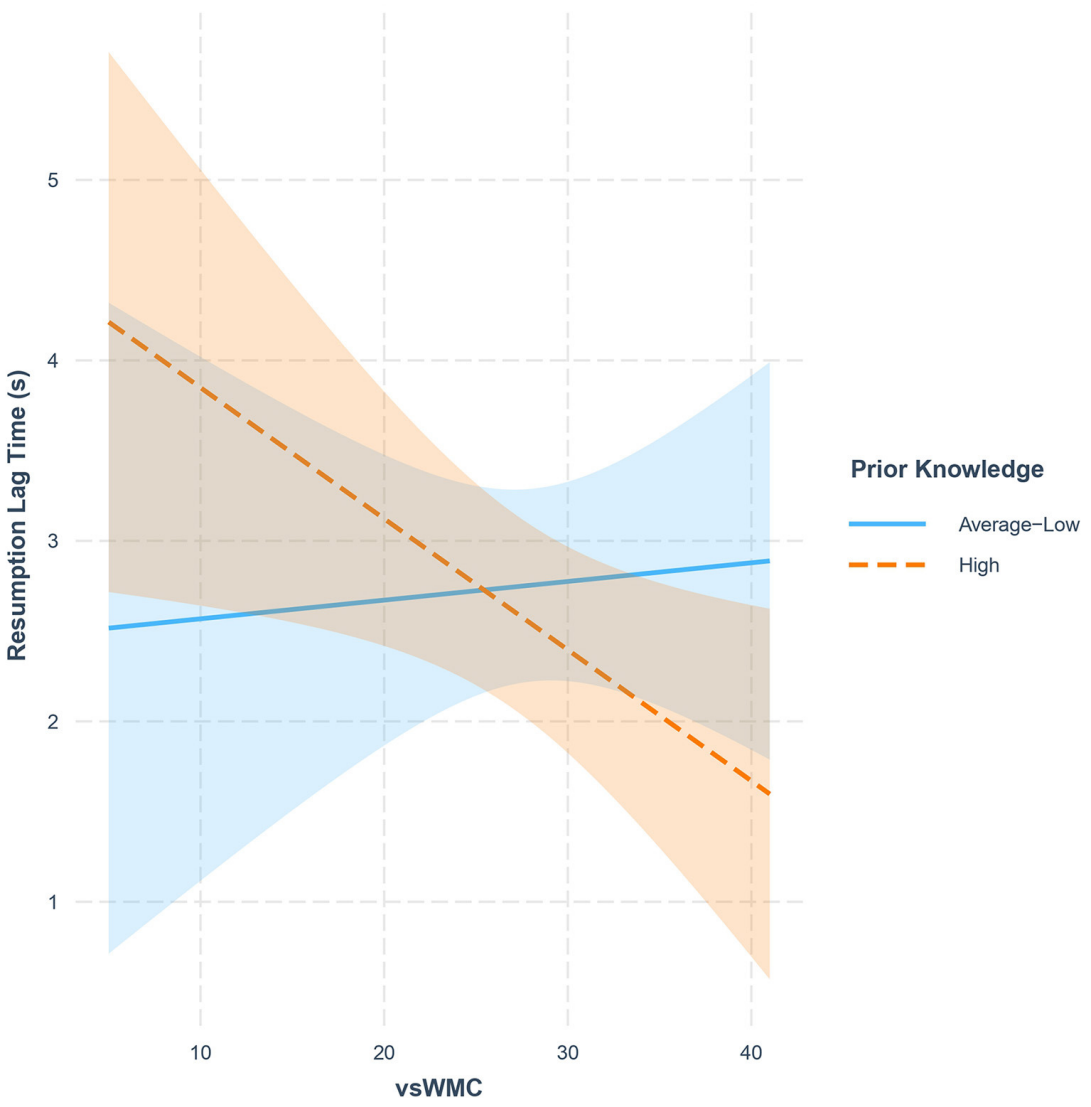
Interaction effect of vsWMC and prior knowledge on resumption lags. Interaction plot showing the effect of vsWMC (x-axis) and resumption lag, in seconds (y-axis), for the average-low (in blue) and high (in orange) prior knowledge groups. The respective bands represent the 95% confidence intervals of the estimates.

The linear mixed-effects model related to our hypotheses operated on *n*_*obs*_ = 272 and obtained a conditional *R*^2^ of 0.23. Taken as individual predictors, vsWMC (β = –0.19, CI = –0.58−0.19, *SE* = 0.19, *t*(43.71) = –0.99, *p* = 0.327) and prior knowledge (β = 0.02, CI = –0.34−0.38, *SE* = 0.18, *t*(43.99) = 0.10, *p* = 0.916) resulted in non-significant contributions. However, the interaction between vsWMC and prior knowledge significantly predicted resumption lags (β = –0.34, CI = −0.65–−0.02, *SE* = 0.16, *t*(45.53) = –2.08, *p* = 0.043). Figure 1 displays variations in the interaction between vsWMC and prior knowledge with respect to the obtained groups. To better understand and visualize these effects, we performed a median split of the prior knowledge score (*Mdn* = 10) and dichotomized our sample into high (*n* = 23, *M* = 16.8, *SD* = 4.56) vs. average-low prior knowledge readers (*n* = 24, *M* = 5.88, *SD* = 2.82), following previous approaches (Altamura et al., 2022; Taub and Azevedo, 2019). Calculating separate Pearson correlations for each subsample, we find a non-significant correlation between vsWMC and resumption lag times for participants with an average-low prior knowledge score [*r*(22)= 0.06, *p* = 0.760]. By contrast, those with high prior knowledge exhibited a non-significant trend toward a moderate negative correlation [*r*(21) = –0.40, *p* = 0.053].

The linear mixed-effects model related to our additional inspections also operated on *n*_*obs*_ = 272 and obtained a conditional *R*^2^ of 0.23. None of the additional predictors under analysis—reading frequency, print vs. digital reading medium, reading comprehension and reading experience—had a significant effect on resumption lag. The effect of the interaction between vsWMC and prior knowledge on resumption lag instead remained significant (*p* = 0.046). Complete results for these analyses can be found in Table 2.

**Table 2:** Results of the linear mixed-effects model for the additional analyses.

**Predictors**	**β**	**95% CI**	***SE***	***t*(*df*)**	***p***
vsWMC	–0.16	[–0.56–0.24]	0.20	–0.78 (43.58)	0.435
Prior knowledge	0.07	[–0.32–0.47]	0.20	0.36 (43.65)	0.717
Medium-frequency readers	–0.23	[–1.21–0.76]	0.49	–0.45 (43.80)	0.652
High-frequency readers	–0.22	[–1.62–1.18]	0.71	–0.30 (44.27)	0.760
Digital reading	0.18	[–0.58–0.94]	0.38	0.46 (43.61)	0.642
Reading comprehension	0.15	[–0.23–0.53]	0.19	0.78 (44.07)	0.433
Reading experience	–0.12	[–0.56–0.32]	0.22	–0.54 (43.65)	0.587
vsWMC: Prior knowledge	–0.32	[–0.64 – –0.01]	0.16	–2.00 (45.50)	0.046

β, standardized estimates; 95% CI, lower and upper limit of 95% confidence intervals; *SE*, standard error; *t*, test statistics; *df*, degrees of freedom; *p*, p values.

## 4 Discussion

Understanding how individual cognitive differences impact how quick we resume reading after interruptions is crucial for optimizing learning and reading efficiency. This study examined the influence of vsWMC and prior knowledge on resumption lags during a reading task interrupted by opinion questions. Specifically, building on current research gaps in the field, our study assesses vsWMC with an independent psychometric test, and addresses the limited data on the multiple dimensions of prior knowledge in interrupted reading. Initially, we hypothesized that higher vsWMC (H1) and greater prior knowledge (H2) would lead to quicker resumption times. Contrary to our hypotheses, these factors did not predict resumption times when considering the inspected factors separately. However, we found a significant interaction between vsWMC and prior knowledge on resumption lag. Further analyses indicated that reading frequency, medium preference, comprehension, and experience were not significant predictors of resumption time, but a significant interaction between vsWMC and prior knowledge was confirmed.

Regarding H1, our findings showed a non-significant effect of vsWMC on resumption lag times. This result aligns with Meys and Sanderson (2013), who also reported a lack of significant effect of spatial memory on recovery time during an interrupted arithmetic task. Werner et al. (2011) instead found a significant relationship between spatial ability scores from a mental rotation task and resumption performance, which became non-significant when considering scores from a paper-folding task. Notably, Meys and Sanderson (2013) pointed out that although some studies have provided evidence for the role of spatial memory and visual search mechanisms in resumption (Cane et al., 2012; Lleras et al., 2005; Ratwani and Trafton, 2008), they were not based on independent psychometric tests of spatial skills. Our observed results provide further support for the limited effect of individual variations in vsWMC on recovery speed. Besides, the reading material used in InteRead belongs to the crime fiction genre, which, with its well-known storylines and the popular figure of Sherlock Holmes, might minimize the need for activating vsWMC. To substantiate this assumption, further experiments are needed that incorporate various genres or literary texts from different time periods. For example, comparing contemporary literature to medieval texts or examining more descriptive passages vs. dynamic ones within the same story could provide valuable insights on whether relying on vsWMC during resumption is mediated by text characteristics.

Regarding H2, our results indicated a non-significant impact of prior knowledge on resumption time, in line with previous work. While Chevet et al. (2022b) showed changes in reading gaze behavior when subjects lack prior knowledge on the text, this effect indeed occurred regardless of the presence of interruptions. Schurer and Schubert (2019) similarly displayed no effect of prior knowledge on attention during reading. These studies, however, only explored prior knowledge in relation to expository texts or paragraphs, with participants being either familiar or unfamiliar with the topic of the text. Interestingly, previous findings also suggested that expository texts would prompt learners to integrate their prior knowledge with learning content more than narrative texts (Wolfe and Woodwyk, 2010). Our findings may indicate that prior knowledge is not a significant cognitive factor for reading resumption and attentional processes, even in narratives. However, further research is necessary to compare expository and narrative texts as well as different reading goals, such as learning or entertainment, within the context of interrupted reading.

Furthermore, we observed that the combined influence of vsWMC and prior knowledge significantly impacts resumption performance. Specifically, readers with high prior knowledge about Sherlock Holmes stories and the crime genre resumed increasingly faster with simultaneously higher vsWMC. Our findings are in line with previous conclusions drawn by Foroughi et al. (2016a), who suggested that readers with low WMC struggle to distinguish between irrelevant and relevant cues upon resumption, resulting in lower performance. They also concluded that readers may need a great familiarity with the particular type and content of a text (i.e., domain-specific expertise) to quickly retrieve information from their long-term working memory (Foroughi et al., 2016a). We postulate that the interaction between vsWMC and prior knowledge may facilitate the resumption process. A stronger integration of vsWMC and prior knowledge might thus allow for discriminating textual information faster upon resumption, retrieving background information more efficiently, resulting in lower resumption times. While the Memory for Goals theory did not consider the role of perceptual processing and the spatial memory component (Ratwani and Trafton, 2008), our results emphasize the necessity of considering both spatial and non-spatial components in understanding the resumption process in reading. In the context of text comprehension and multimedia learning, these findings support theoretical integration between prior knowledge and visuo-spatial processing (Schnotz, 2002). More precisely, readers with high prior knowledge and greater vsWMC are likely more skillful at constructing and storing mental models of the text, facilitating easier reactivation upon resumption. Although visually highlighting the word where an interruption occurred significantly sustains resumption (Cane et al., 2012; Jo et al., 2015), future research should investigate how this support varies while considering individual differences in prior knowledge and vsWMC. Such insights could enhance the design of intelligent tutoring systems and reading applications, providing personalized resumption support. Our results may also suggest that educators should integrate visuo-spatial skills, often insufficiently fostered in classrooms (Mathewson, 1999), with traditional text comprehension training to improve students' resumption performance during reading.

Our additional analyses indicated that reading frequency, reading medium, reading comprehension and reading experience did not significantly predict resumption performance. The non-significant effect of reading frequency and medium is in agreement with previous preliminary research showing that print and digital reading weekly hours had no significant correlation with gaze behavior during resumption (Altamura et al., 2022). While most prior studies showed no evidence of interruptions negatively impacting comprehension, a few studies also found both negative and positive effects (Chevet et al., 2022a). However, prior work evaluating comprehension during interrupted reading either designed extensive questionnaires (Chevet et al., 2022a) or used well-established reading comprehension tasks such as the SAT (Foroughi et al., 2015). The short questionnaire underlying the InteRead dataset might be not sufficiently reliable for a thorough assessment of reading comprehension. Regarding the perceived reading experience instead, similar results are found in Chevet et al. (2022c), where the relation between attentional disruption and enjoyment of the reading experience lacks significant impacts. Nevertheless, our additional analyses confirmed the strong predictive power of the interaction between vsWMC and prior knowledge in determining resumption speed.

Limitations of this research include reliance on a single dataset, hence a limited generalizability to different materials. Future work could extend the current investigation with more diverse samples, reading material and assessment metrics as well as explore additional factors that may impact resumption behavior. The type of interruption may also represent a limitation. Although the interrupting task used in InteRead aimed to minimize the potential for subvocal rehearsal, previous research has utilized various types of interruptions, often yielding different results (Cauchard et al., 2012; Katidioti et al., 2016; Wirzberger and Rey, 2018). Future research should further investigate how different interruption types affect reading resumption times. Lastly, InteRead relies on manual annotation of gaze data for detecting resumption lags (Zermiani et al., 2024). This could be limited by subjective variations among raters. Implementing and evaluating gaze-based resumption detection algorithms may enhance reliability and objectivity in future studies.

In conclusion, the presented work contributes to further understanding how individual differences in cognitive factors, such as vsWMC and prior knowledge, impact the time required to recover from interruptions during reading. These insights have significant implications for the design of adaptive learning technologies and educational interventions tailored to individual needs. Our findings underscore the complex relationship between these cognitive factors and reading resumption performance, suggesting potential future research and practical applications in education.

## Data Availability

Publicly available datasets were analyzed in this study. This data can be found here: https://doi.org/10.17605/OSF.IO/43J5F.

## Footnotes

^1^Version 1.1.28.

^2^Version 3.1.3.
